# Key genes and immune infiltration in chronic spontaneous urticaria: a study of bioinformatics and systems biology

**DOI:** 10.3389/fimmu.2023.1279139

**Published:** 2023-11-15

**Authors:** Wenxing Su, Yu Tian, Yuqian Wei, Fei Hao, Jiang Ji

**Affiliations:** ^1^Department of Dermatology, The Second Affiliated Hospital of Soochow University, Suzhou, China; ^2^Dermatology and Plastic Surgery Center, The Third Affiliated Hospital of Chongqing Medical University, Chongqing, China; ^3^Department of Dermatology, The First Affiliated Hospital of Chengdu Medical College, Chengdu, China; ^4^Department of Dermatology, Nantong Third People’s Hospital, Nantong, China

**Keywords:** chronic spontaneous urticaria, bioinformatics, differentially expressed genes, hub genes, pathway, immune infiltration, TNF, NF-κB

## Abstract

**Background:**

Chronic spontaneous urticaria (CSU) is defined by the spontaneous occurrence of wheals and/or angioedema for >6 weeks. The pathogenesis involves skin mast cells, but the complex causes of their activation remain to be characterized in detail.

**Objectives:**

To explore disease-driving genes and biological pathways in CSU.

**Methods:**

Two microarray data sets, e.g., GSE57178 and GSE72540, with mRNA information of skin from CSU patients, were downloaded from the Gene Expression Omnibus (GEO) database. An integrated bioinformatics pipeline including identification of differentially expressed genes (DEGs), functional enrichment analysis, protein-protein interaction (PPI) network analysis, co-expression and drug prediction analysis, and immune and stromal cells deconvolution analyses were applied to identify hub genes and key drivers of CSU pathogenesis.

**Results:**

In total, we identified 92 up-regulated and 7 down-regulated genes in CSU lesions. These were significantly enriched in CSU-related pathways such as TNF, NF-κB, and JAK-STAT signaling. Based on PPI network modeling, four genes, i.e., IL-6, TLR-4, ICAM-1, and PTGS-2, were computationally identified as key pathogenic players in CSU. Immune infiltration analyses indicated that dendritic cells, Th2 cells, mast cells, megakaryocyte-erythroid progenitor, preadipocytes, and M1 macrophages were increased in lesional CSU skin.

**Conclusion:**

Our results offer new insights on the pathogenesis of CSU and suggest that TNF, NF-κB, JAK-STAT, IL-6, TLR-4, ICAM-1, and PTGS-2 may be candidate targets for novel CSU treatments.

## Introduction

CSU is usually characterized by typically short-lived, itchy, and fleeting wheals, angioedema, or both, occurring spontaneously for longer than 6 weeks (1). CSU affects about 1-4% of the world population, across all ages, mostly young and middle-aged women (2). CSU carries a significant emotional and economic burden for the patient (3),often lasting a long time and affecting many areas of daily life. To date, only antihistamines and omalizumab are licensed for the treatment of CSU, but there are still many patients who do not use these therapies and need more effective treatment.

The development of novel and better treatments for CSU is very much needed. Possible pathophysiological mechanisms now known for CSU include autoantibodies and other mast cell activation signals that lead to skin MC degranulation, release of pro-inflammatory mediators, and recruitment of inflammatory cells to the skin. Beyond this, the pathogenesis of urticaria is poorly understood (4–6). Insights into the complexities and drivers of the pathogenesis of CSU can point to promising targets of newer and more efficacious targeted biological treatments for severe, refractory CSU.

Microarray analysis can detect thousands of gene expression changes in a short time (7). Bioinformatic analyses of microarray gene expression datasets can identify DEGs and relevant functional pathways (8–10). As of now, two CSU studies have assessed skin gene expression (11–13), but joint analyses of these microarray datasets, GSE57178 and GSE72540 are limited. One recent study of these datasets recently reported that the IL-6/miR-149-5p/ZBTB20-AS1 axis may be involved in mast cell activation in CSU (14). The lesional immune cell infiltrate and core genes, however have not been investigated by bioinformatics analyses.

In this study, we performed deep bioinformatic analyses of the merged CSU microarray datasets GSE57178 and GSE72540 available from the GEO, which contain cutaneous gene expression data from 16 CSU patients and 13 healthy individuals. The aim of this study was to identify key genes and immune cells related to the pathological mechanism of CSU.

## Methods

### Microarray studies and datasets from GEO

Two microarray datasets (GSE57178 (11) and GSE72540 (12)) were downloaded from GEO (http://www.ncbi.nlm.nih.gov/geo). The details are shown in **Table 1**. GSE57178 contains the mRNA information of lesional skin (N=6) and non-lesional skin (N=7) of CSU patients, and of skin from healthy individuals (N=5). GSE72540 also contains information on lesional CSU skin (N=10), non-lesional CSU skin (N=13), and the skin of healthy control subjects (N=8). All included patients showed active CSU, i.e. urticaria activity score 7 (UAS7) ≥ 11 for at least 3 months, and were refractory to antihistamine treatment.

**Table 1:** Details of the GEO datasets. Lesional skin (LS), non-lesional skin (NL), normal skin (NS).

Dataset	Platform	No. of Samples (LS, NL, NS)	References
GSE57178	GPL6244 Platforms (Affymetrix Human Gene 1.0 ST Array)	6,7,5	Patel OP et al. (11)
GSE72540	GPL16699 Platforms (Agilent-039494 SurePrint G3 Human GE v2 8x60K Microarray 039381)	10,13,8	Giménez-Arnau A et al. (12)

### Data preprocessing and screening for differentially expressed genes

Background correction and data normalization were completed through the “Affy” package and the RMA algorithm.The limma package was applied to identify the DEGs between CSU skin and the skin of healthy controls. |LogFC| > 1 and P-value < 0.05 were defined as the cut-off.

### Enrichment analyses

GSEA (Gene Set Enrichment Analysis) is a calculation method to determine whether a set of genes defined in advance are differentially expressed in different samples (15). In order to explore whether most of the genes in the predefined gene set are highly expressed or poorly expressed in the sample, the H. All. V6.2. Symbols.gmt [Hallmarks] gene set database in GESA software is used for analysis. Significantly enriched biological process (BP) items of the DEGs were obtained by DAVID (version 6.8) and Metascape. Biological pathways analysis was completed by the functional enrichment analysis tool (Funrich) (16). The pathway enrichment analyses of DEGs were evaluated by two databases, including “KEGG pathway” and “Reactome”.

### PPI network construction and modules analysis

The PPI network of DEGs was predicted by STRING (version 11.0; http://string-db.org) and visualized by Cytoscape (version 3.7.2). Key modules were identified by using The Molecular Complex Detection (MCODE; version 1.5.1) of Cytoscape (MCODE scores > 5, degree cut‐off = 2, node score cut‐off = 0.2, max depth = 100 and k‐score = 2).

### Identification of hub genes

The cytoHubba plug-in in the Cytoscape software was used to analyze the topological attributes of nodes in the network, and the parameters are set to no weight. By sorting the score of each node, we identified the important nodes involved in protein interaction in the network. We selected six commonly used parameters to calculate the top ten genes and their respective rankings and obtained the common hub genes through the Venn diagram. GeneMANIA (http://www.genemania.org/) was used to explore the co-expression genes and functions of hub genes. The Drug-Gene Interaction database (DGIdb 3.0; http://www.dgidb.org/) was used to discover the existing targeted drugs for hub genes. All the network map was formed by Cytoscape.

### Immune and stromal cells deconvolution analyses

xCell is an evaluation tool of immune cell infiltration based on gene expression data, which can identify potential immune and stromal cell subsets and calculate their relative abundance in tissues (17). We evaluated the immune cell infiltration of two microarray data by xCell. Finally, their common differential immune and stromal cells were obtained by Venn diagrams.

### Correlation analysis of hub genes and infiltrating immune cells

Spearman correlation analysis was used to explore the relationship between hub genes and infiltrating immune cells. The results were visualized by using the “ggplot2” software package.

## Results

### In CSU, gene expression signatures of lesional skin are different and distinct from healthy skin

**Figure 1:**
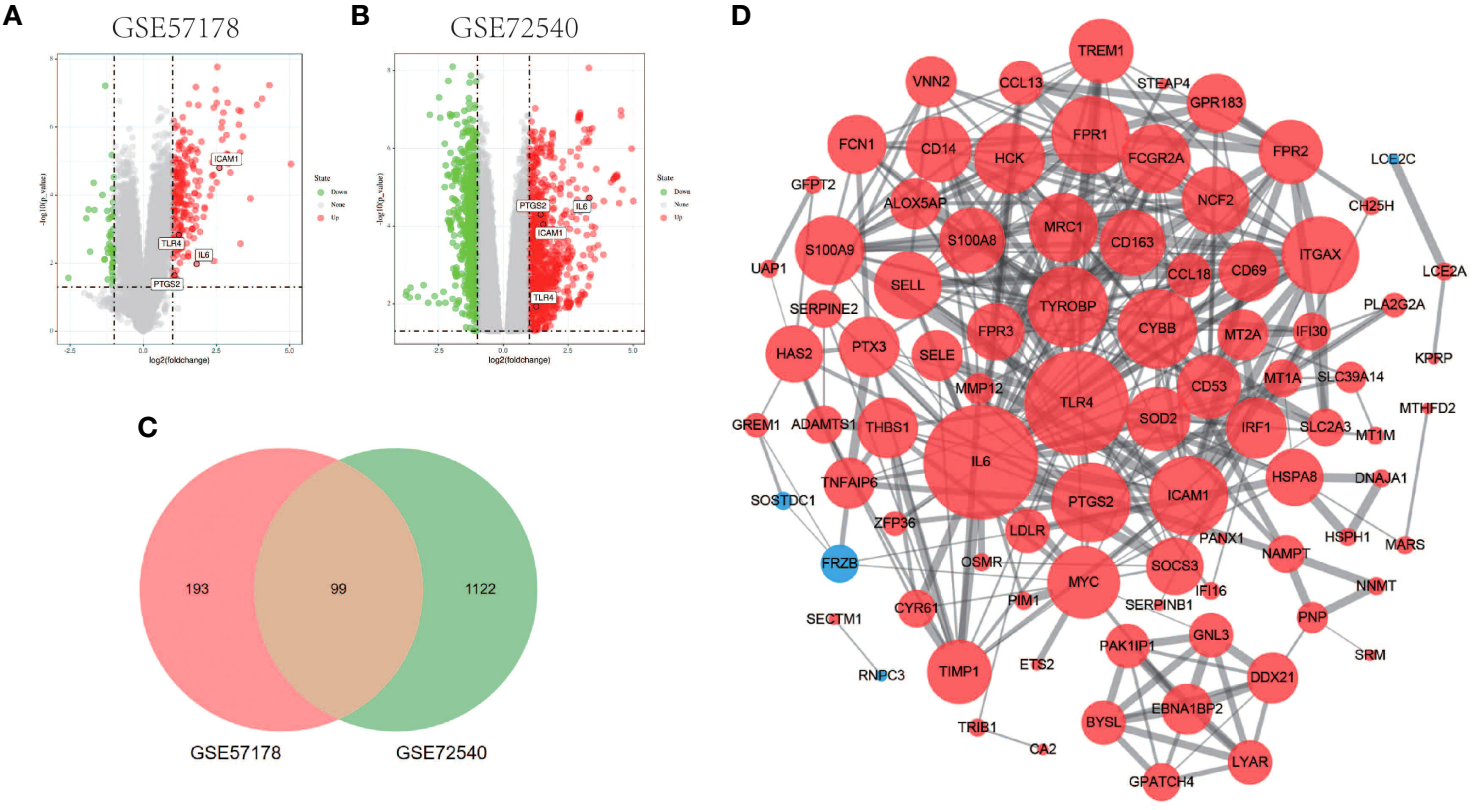
Volcano figure, Venn diagram, PPI network of common DEGs. **(A, B)** Volcano plot of DEGs. Among them, red indicates up-regulated genes and green indicates down-regulated genes. Gray dots represent genes with no significant difference. **(C)** GSE57178 and GSE72540 have 99 DEGs in common. **(D)** PPI network is constructed according to 99 common DEGs, in which up-regulated genes are represented by red circles and down-regulated genes by blue circles. The size of the gray node represents the size of the topological properties of the corresponding protein in the network, and the thickness of the line represents the strength of evidence of interaction between the two proteins.

**Figure 2:**
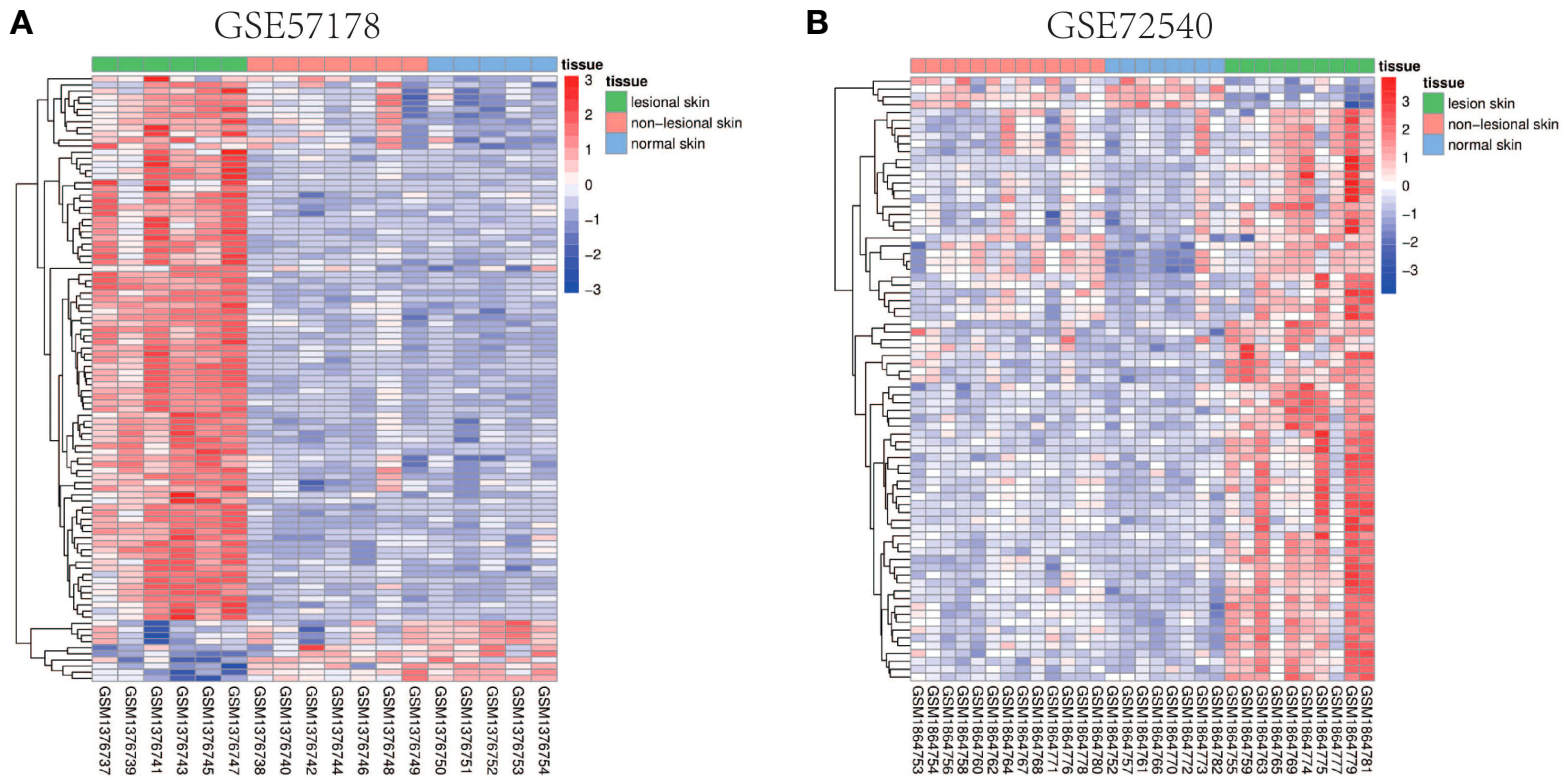
Heat map of common DEGs. **(A, B)** Heat maps of common DEGs between GSE57178 and GSE72540. Up-regulated genes are shown in red, down-regulated genes are shown in blue, and genes with little change are shown in white.

Comparing lesional CSU skin and healthy control samples, 292 and 1221 DEGs were identified in GSE57178 and GSE72540, respectively, of which 99 were in both (**Figure 1**), 92 upregulated and 7 downregulated DEGs (**Figure 1**). The heat map shows that these DEGs can basically distinguish CSU lesion samples from healthy control samples (**Figure 2**).

### In CSU lesions, gene sets of TNF/NF-κB, IL-6/JAK/STAT3 and IFN-γ pathways are enriched

**Figure 3:**
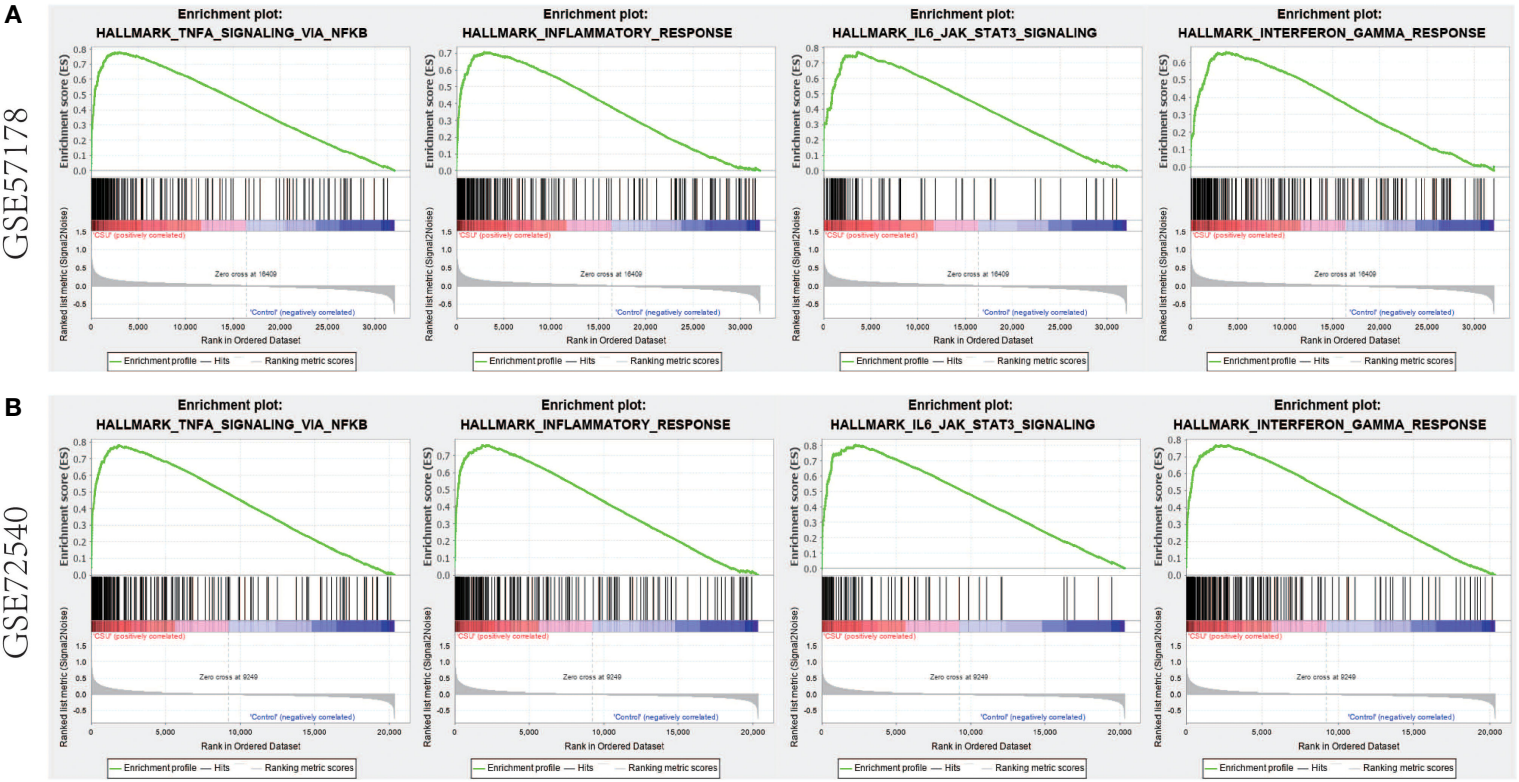
GSEA enrichment analysis. **(A, B)** The common gene set pathway obtained by GESA analysis of GSE57178 and GSE72540 respectively.

**Figure 4:**
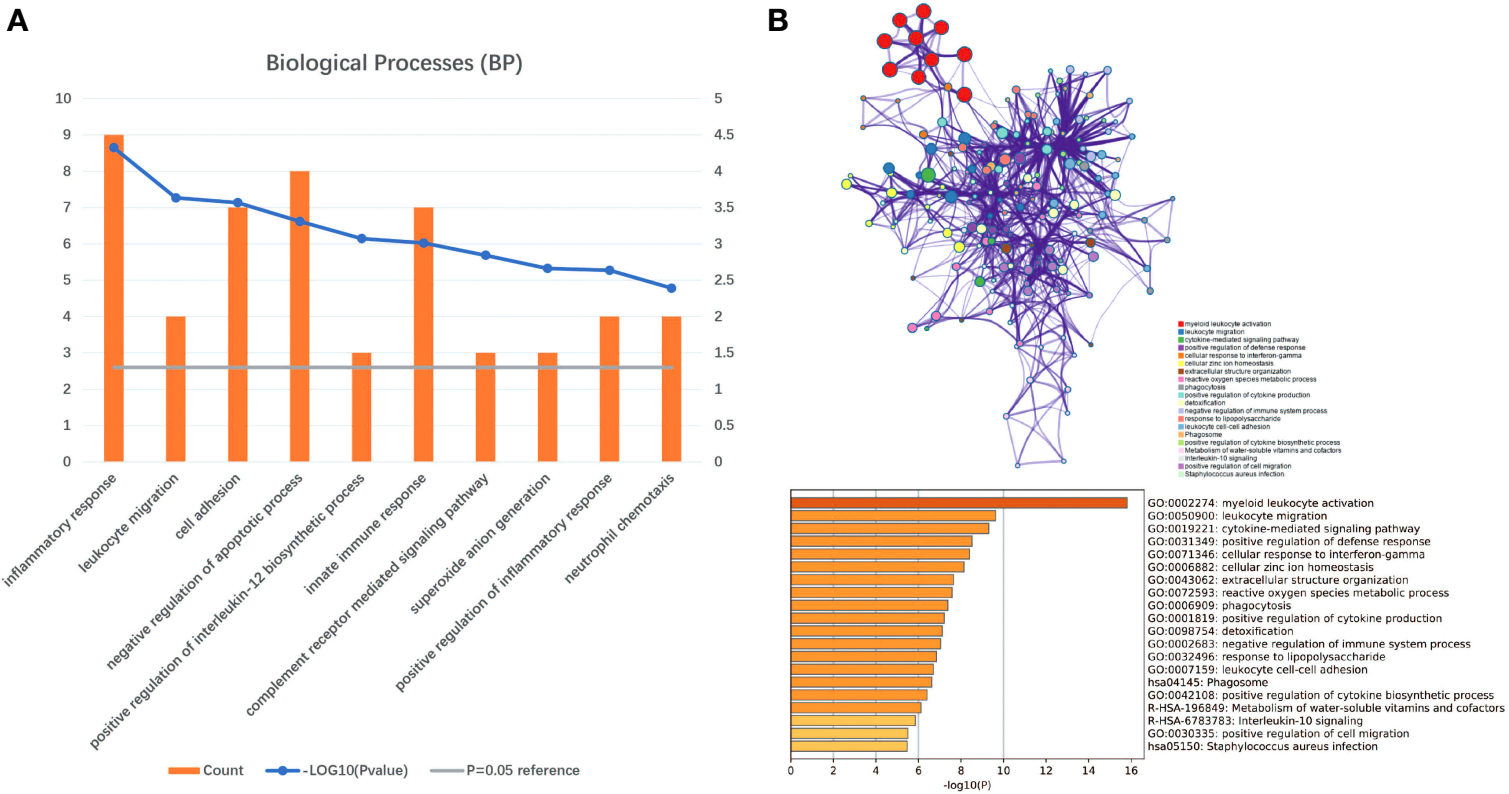
Significant biological processes were obtained through DAVID and Metascape. **(A)** The top 10 biological processes form DAVID. **(B)** The top 20 biological processes enriched by Metascape.

**Figure 5:**
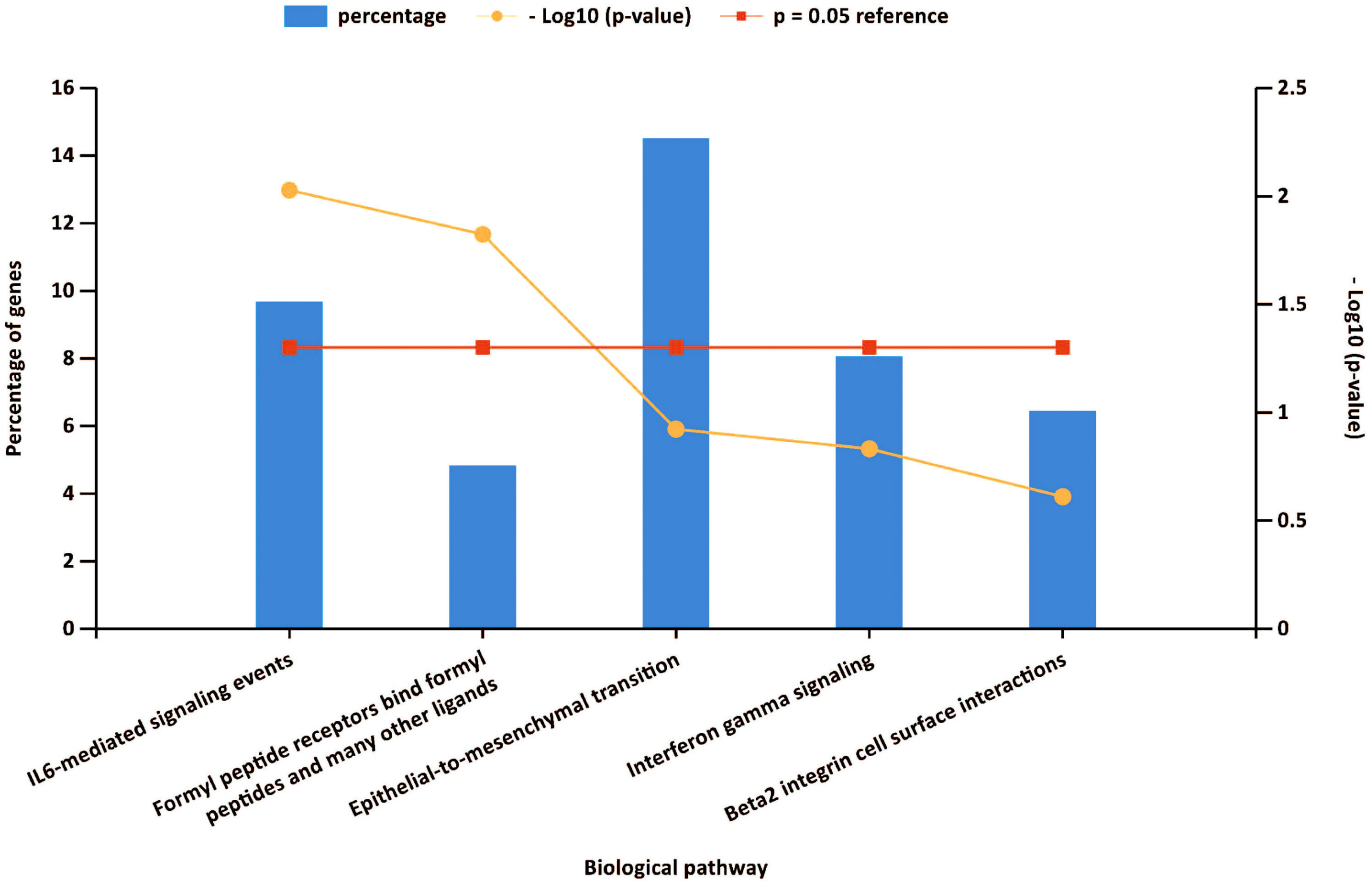
Funrich analysis of DEGs. The first five biological pathways enriched by Funrich software.

**Figure 6:**
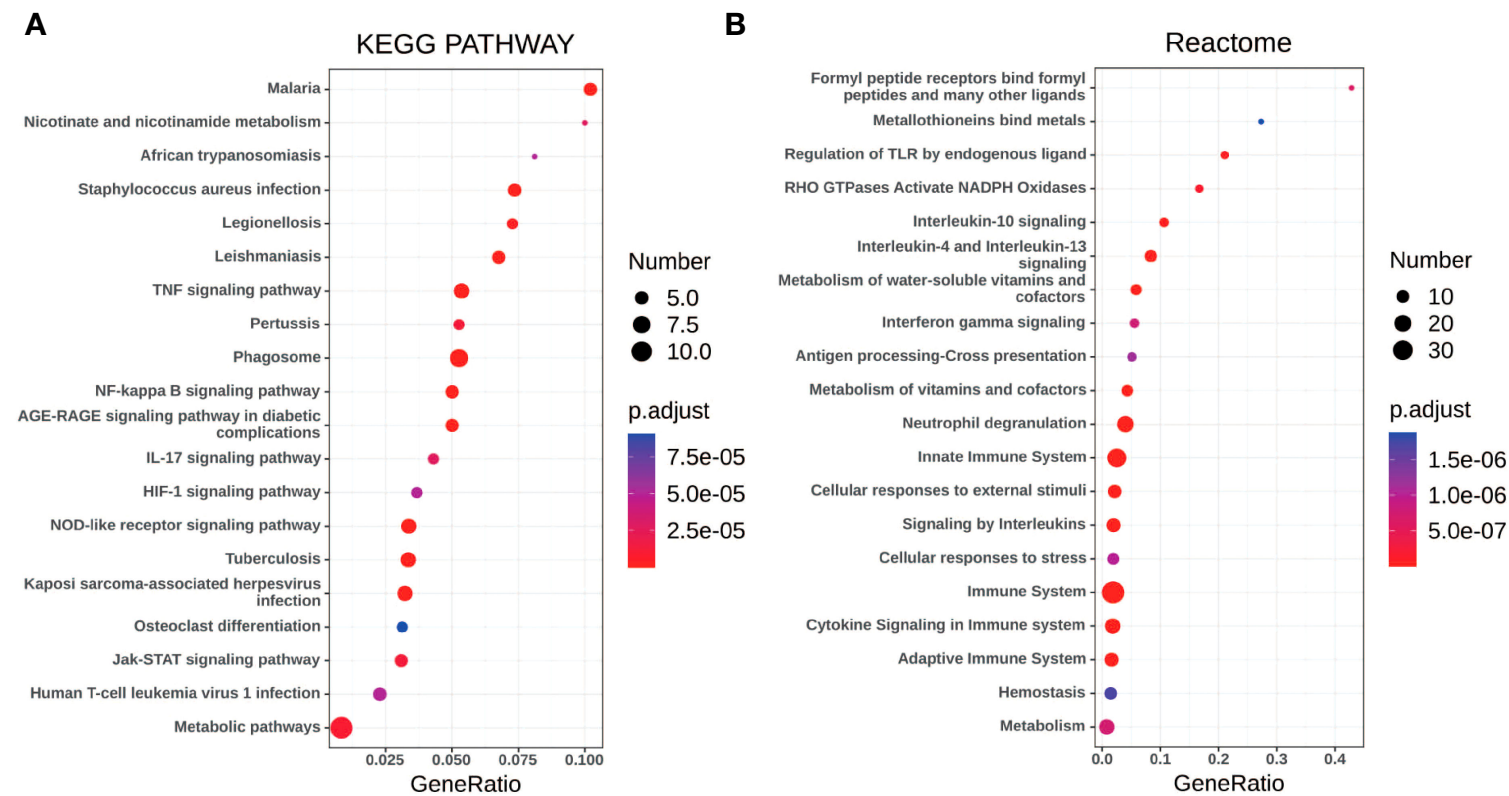
The pathway analysis of DEGs by KOBAS 3.0. **(A, B)** The pathway analysis of all the DEGs via KEGG and Reactome database. The size of the dot represents the number of enriched genes, the color represents the size of the P-value, the abscissa represents the proportion of the enriched genes to the total number of gene sets, and the ordinate represents the name of the pathway.

Gene set enrichment analyses showed that four common gene sets were significantly enriched in CSU and healthy controls samples (**Figure 3**), including TNF-mediated NF*-κB* pathway, IL-6/JAK/STAT3 signaling pathway, and responses to interferon-gamma. Biological processes enrichment analysis indicated that these were mainly linked to inflammation and leukocyte migration (**Figure 4** and **Table S1**). Enrichment analyses of biological pathways demonstrated that DEGs in lesional vs non lesional skin were mainly enriched in IL-6-mediated signaling events and the biological process by which formyl peptide receptors bind to formyl peptides and many other ligands (**Figure 5**). Moreover, our KEGG analysis showed that 4 pathways were significantly different in lesional vs healthy skin of the control group, including Phagosome, Malaria, TNF signaling pathway, and Staphylococcus aureus infection. Reactome enrichment analyses revealed that the most significantly enriched pathways in CSU lesions vs non lesional skin were Immune System, Innate Immune System, Neutrophil degranulation, and Interleukin-4 and Interleukin-13 signaling (**Figures 6A, B**, **Tables S2 and S3**).

### Development of CSU lesions is linked to TNF, NF-κB, and JAK-STAT signaling pathways

**Figure 7:**
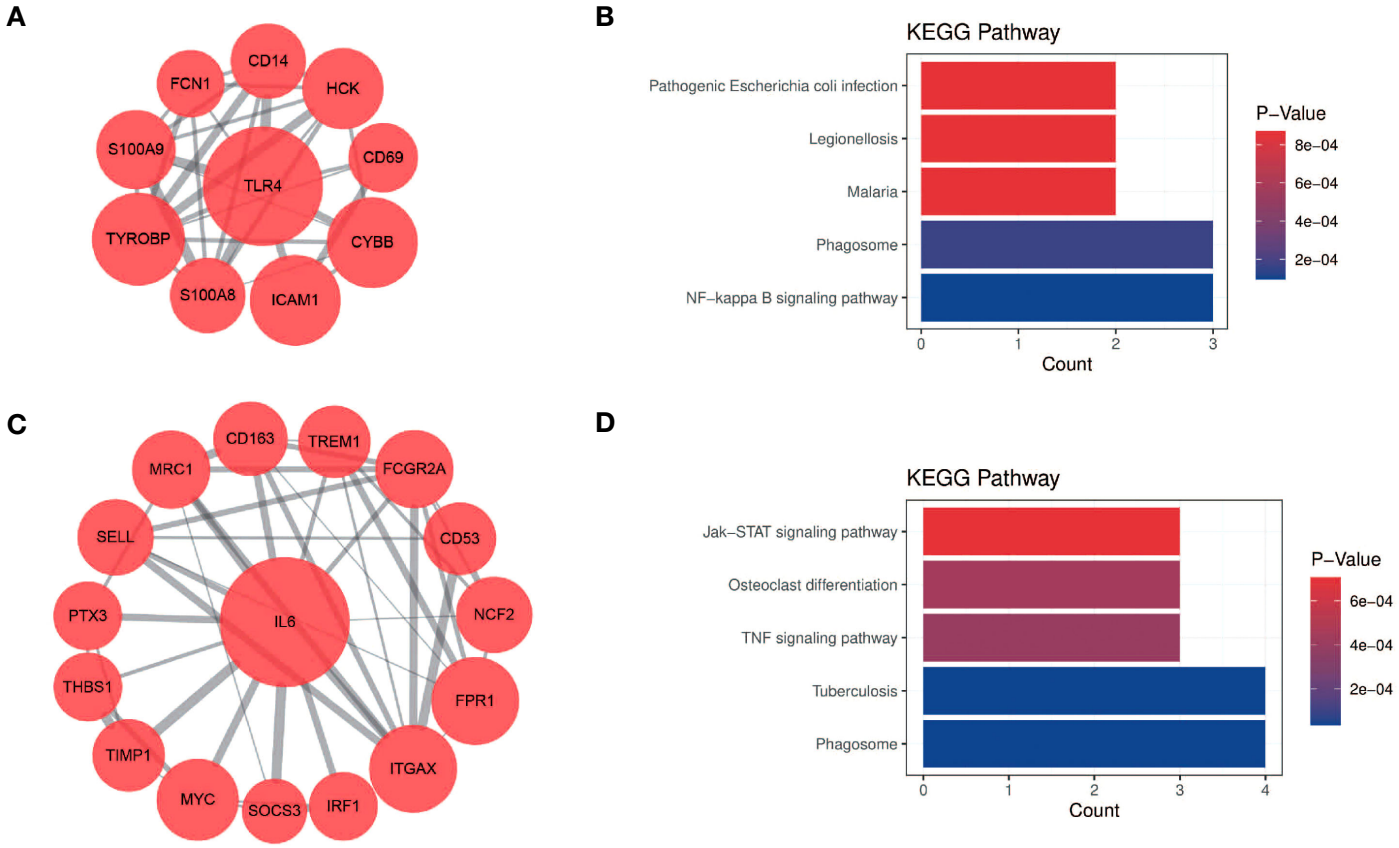
KEGG pathway analysis of modular genes. **(A, B)** Module 1 and the KEGG pathway enrichment analysis. **(C, D)** Module 2 and the KEGG pathway enrichment analysis.

The PPI network of DEGs generated by Cytoscape consists of 87 nodes and 347 interaction pairs (**Figure 1D**). Two important gene modules were generated by MCODE plug-ins (**Figures 7A, C**). KEGG pathway enrichment analysis showed that three pathways are closely related to CSU, including TNF signaling pathway, NF-κB signaling pathway and JAK-STAT signaling pathway (**Figure 7B, D**).

### Four hub genes IL-6, TLR-4, ICAM-1, and PTGS-2 are significantly linked to CSU lesion formation

**Figure 8:**
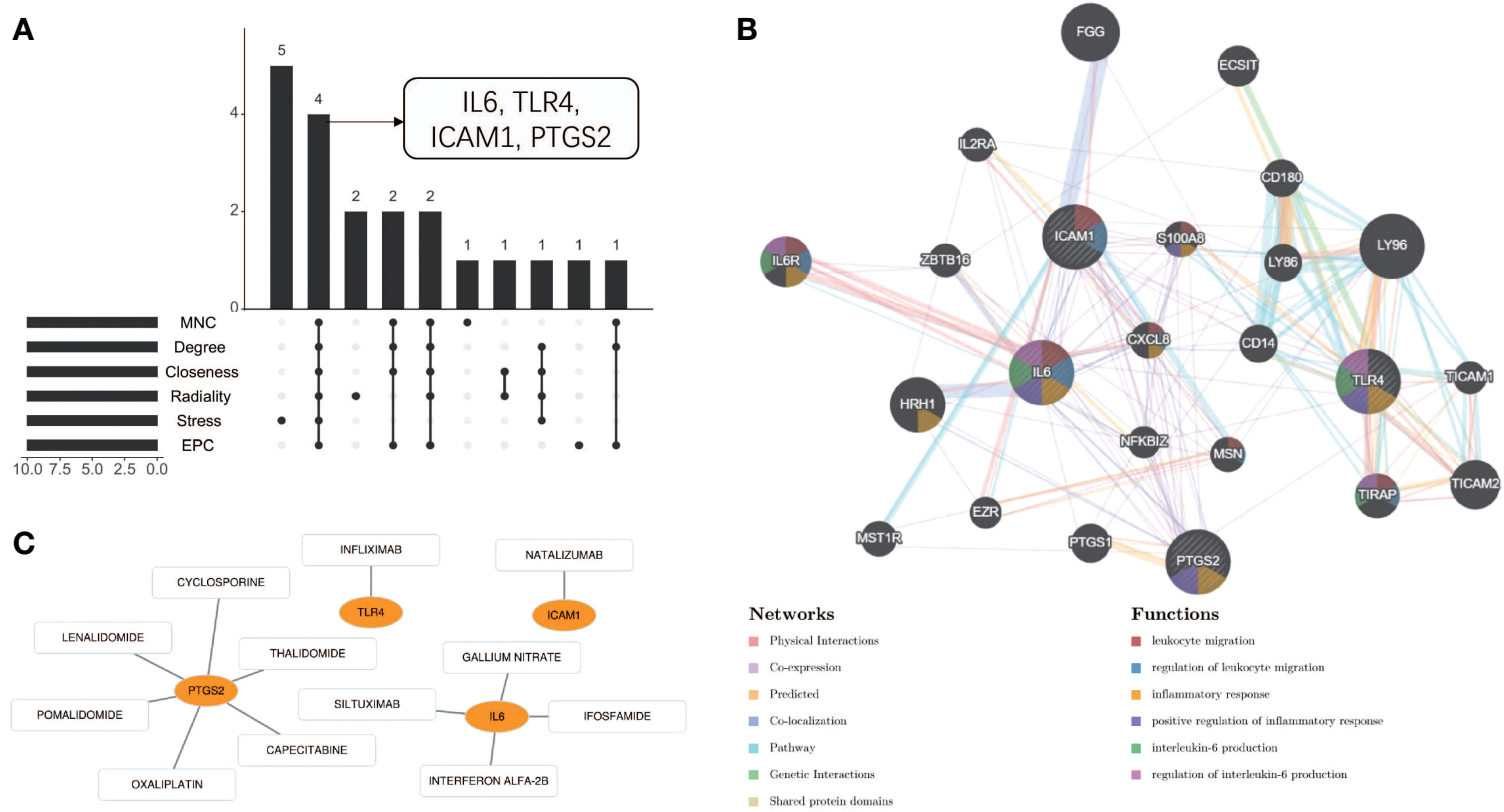
Co-expression analysis and drug-gene interaction analysis of hub genes. **(A)** Four common hub genes obtained by six algorithms. **(B)** The network diagram of Hub genes and their co-expressed genes. **(C)** Drug-gene interaction diagram.

Using the 6 algorithms (MNC, Radiality, Stress, Degree, Closeness, and EPC) in the cytoHubba plugin, we obtained 4 lesional skin-expressed hub genes: IL-6, TLR-4, ICAM-1, and PTGS-2 (**Figure 8A** and **Table 2**). Then, a network of these 4 common hub genes and their co-expression genes were analyzed by GeneMANIA online platform. The analysis of co-expression network showed that hub genes and co-expression genes were mainly involved in the process of leukocyte migration and inflammatory response (**Figure 8B**). Furthermore, we obtained 12 drugs (FDA‐listed + Immunotherapies) that paired with these hub genes from the DGIdb (**Figure 8C**).

**Table 2:** Top 10 hub genes among the six algorithms.

MNC	Degree	Closeness	Radiality	Stress	EPC
HCK	HCK	FPR1	IRF1	HSPA8	HCK
FPR1	FPR1	PTGS2	PTGS2	PTGS2	FCGR2A
PTGS2	PTGS2	ITGAX	TIMP1	ICAM1	FPR1
ITGAX	ITGAX	ICAM1	IL6	IL6	PTGS2
IL6	IL6	IL6	ICAM1	EBNA1BP2	ITGAX
ICAM1	ICAM1	MYC	MYC	LDLR	IL6
MRC1	MYC	TLR4	TLR4	GNL3	ICAM1
TLR4	TLR4	SOD2	SOD2	MYC	TLR4
CYBB	CYBB	CYBB	CYBB	TLR4	CYBB
TYROBP	TYROBP	TYROBP	TYROBP	HAS2	TYROBP

### The spectrum of infiltrating cells in CSU lesions is different from healthy skin

**Figure 9:**
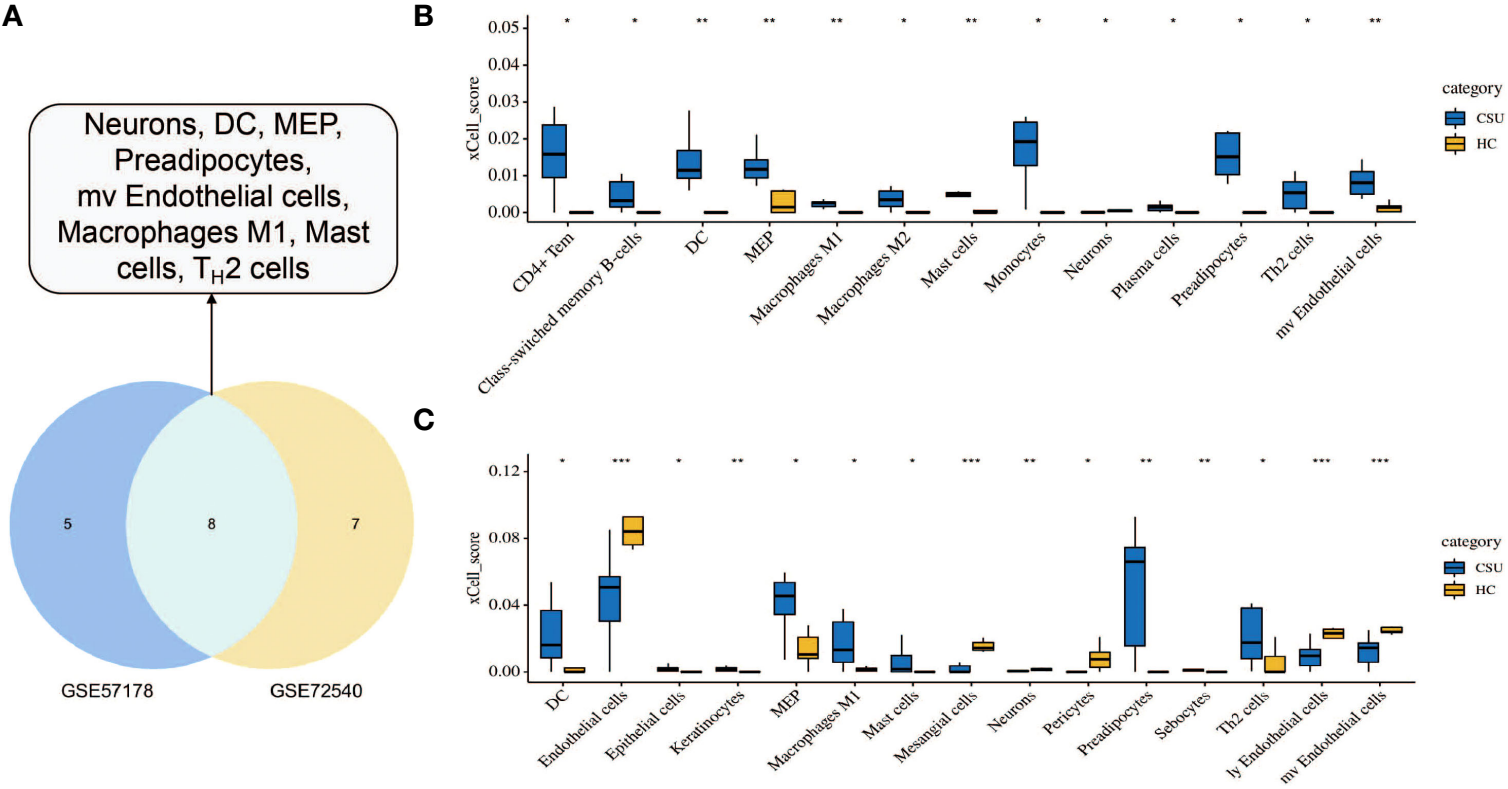
Immunological and stromal cell infiltration analysis. **(A)** Common differential cell types in GSE57178 and GSE72540. XCell scores of 64 cell types in **(B)** GSE57178 and **(C)** GSE72540.

To investigate the immune cell types that may be infiltrated in CSU lesions, cell enrichment analyses done with xCell showed marked differences between CSU and healthy controls samples in both datasets, GSE57178 and GSE72540. Eight cell types showed much higher expression in lesional CSU skin: immune cells, sensory nerves, dendritic cells (DC), preadipocytes, megakaryocyte-erythroid progenitor (MEP), mast cells, M1 macrophages, microvascular (mv) endothelial cells, and Th2 cells (**Figure 9A**). Specifically, both in GSE57178 and GSE72540, compared with normal healthy skin, CSU lesions showed higher infiltrations of DC, preadipocytes, Th2 cells, MEP, mast cells, and macrophages M1(**Figure 9B, C**), respectively.

### Lesional CSU hub genes correlate with immune and stromal cell infiltration

**Figure 10:**
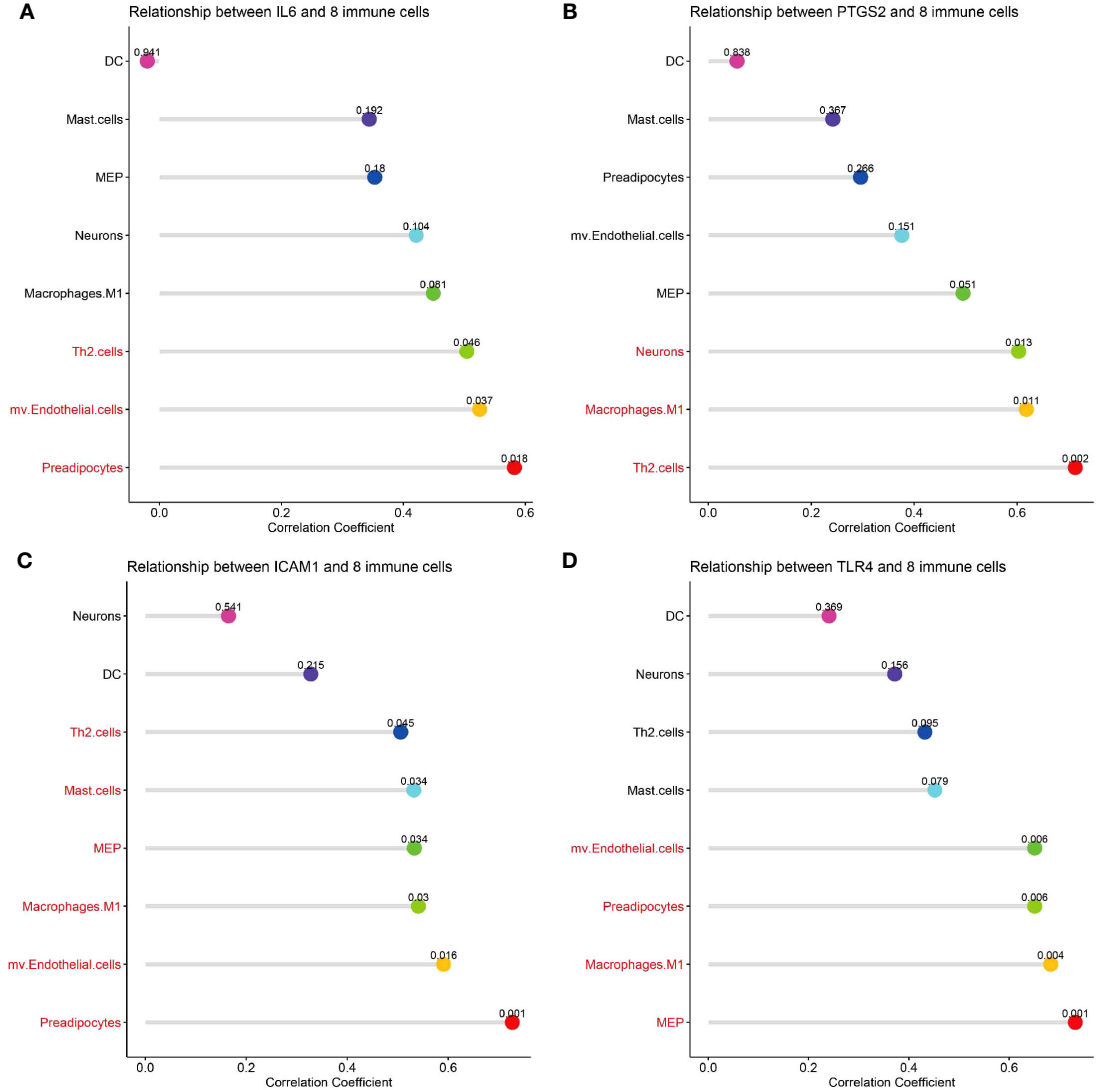
Correlation analysis of hub genes with immune and stromal cells. **(A-D)** The correlation between common differential cell types and IL-6, PTGS-2, ICAM-1, TLR-4. The terms marked with red indicate the cells that are related to the hub gene.

Correlation analysis between hub genes and infiltrating immune cells in CSU lesions showed that 1) IL-6 (**Figure 10A**) was positively correlated with activated Th2 cells, preadipocytes, and mv Endothelial cells; 2) PTGS-2 (**Figure 10B**) was positively correlated with Th2 cells, neurons, and macrophages M1; 3) ICAM-1 (**Figure 10C**) was positively correlated with Th2 cells, mast cells, macrophages M1, mv Endothelial cells, MEP, and preadipocytes; 4) TLR-4 (**Figure 10D**) was positively correlated with mv Endothelial cells, MEP, macrophages M1, and preadipocytes.

## Discussion

This study investigated potential disease driving gene signatures and functional networks in patients with CSU through microarray analysis. In total, 99 DEGs including 92 up-regulated genes and 7 down-regulated genes between CSU and healthy controls were identified. By enrichment analysis we identified core modules and three signaling pathways i.e. TNF, NF-κB, and JAK-STAT, that are closely related to the development of CSUs. In addition, we identified four hub genes, including IL-6, TLR-4, ICAM-1, and PTGS2.In addition, xCell is often used to assess local immune cell infiltration in skin biopsies of CSU patients. CSU lesions contained higher proportions of DCs, Th2 cells, MCs, megakaryocyte-erythroid progenitors, preadipocytes, and M1 macrophages compared to healthy skin. Finally, the above four hub genes IL-6, TLR-4, ICAM-1, and PTGS2 were found to be correlate with immune and stromal cell infiltration in CSU lesions.

### TNF is a key inflammatory mediator in CSU inflammation

CSU is not only associated with autoimmune processes but also systemic inflammatory responses (18). The release of various inflammatory mediators (histamine, protease, leukotriene and TNF, etc.) is closely related to the wheals of CSU. TNF, which also can be secreted by T cells, natural killer (NK) cells, monocytes, macrophages, basophils, eosinophils, keratinocytes, and fibroblasts, et al. (19). Dysregulation of the TNF pathway is a key feature and causative factor in many autoimmune and inflammatory diseases (20, 21). The formation of wheals in CSU is closely related to the activation of TNF signaling pathway, marked by 1) increased circulating concentrations of TNF-R, sTNF-R1, and sTNF-R2, 2) serum TNF level was positively correlated with disease activity in the samples of patients with CSU, 3) TNF is upregulated in the lesional and non-lesional skin of patients with CSU (22, 23). In addition, the use of TNF inhibitors (Etanercept, Adalimumab, Infliximab) for the treatment of CSU have also been validated in clinical care (24). Consistent with these findings, in the present study, we also observed that TNF is a hub gene of CSU. In sum, all these findings provide a theoretical basis for the potential of TNF targets in the treatment of CSU. Building on these foundations, larger and more rigorous trials are needed to make a stronger case that TNF for CSU is safe and effective.

### NF-κB activation may link to chronic inflammation in CSU

CSU is also associated with chronic inflammation. And most chronic diseases, caused by lifestyle factors, appear to be linked to inflammation (25). As we all know, NF-κB is a pivotal pathway that mediates the pathogenesis of inflammatory skin diseases (26). When cells are subjected to various intracellular and extracellular stimuli, NF-κB dimers are released, which are further activated and translocated to the nucleus, leading to the production of inflammatory cytokines such as IL-6 and TNF (27, 28). In turn, these inflammatory cytokines promote the activation of NF-κB signalling, leading to a complex inflammatory cascade *in vivo (* 27). In the present study, we have demonstrated that both IL-6 and TNF are hub genes of CSU. Besides, our enrichment analysis results showed that the NF-κB signaling pathway was significantly related to the occurrence and development of CSU. Based on these findings, we speculate that IL-6/TNF-mediated over-activation of the NF-κB signaling pathway is a new therapeutic target for CSU.

### JAK/STAT3 signaling pathway involves in inflammatory reactions and allergic responses of CSU

It has been shown in recent years that the JAK/STAT3 signalling pathway is associated with the pathogenesis of CSU. In the lesions of CSU patients, the high expression of OSMR gene can promote the expression of JAK/STAT3 signaling pathway related genes (29). They further observed that OSMR gene silencing can significantly inhibit the activity of JAK/STAT3 signal pathway, promote cell proliferation and migration, and further inhibit epithelial cell apoptosis in a chronic autoimmune urticaria mouse model. Other studies have also found that IL-9 and IL-10 can promote CSU through activation of the JAK/STAT3 signalling pathway (30). Our GSEA enrichment analysis based on both datasets showed that the IL-6/JAK/STAT3 pathway was also highly expressed in the CSU patient group. Additionally,the IL-6/JAK/STAT3 signalling pathway has been shown to be associated with mast cell degranulation and allergic reactions (31). All aforementioned findings indicate that blocking the IL-6/JAK/STAT3 signalling pathway may be a novel approach for the treatment of CSU.

### IL-6, the interleukin related with CSU

IL-6 is an important regulator of inflammation and immune responses and is associated with a variety of autoimmune and chronic inflammatory diseases (32–34). Although the imbalance of inflammatory cells was detected in skin biopsies of CSU patients, the source of IL-6 in CSU has not been identified (6, 35). Previously, several studies have reported higher IL-6 levels in patients with CSU compared to those observed in the healthy group (36–39). IL-6 levels could be linked to CSU disease activity (40). In addition, IL-6 levels in CSU patients were reduced by omalizumab therapy.

Besides, IL-6/soluble IL-6 receptor (sIL-6R) complex can stimulate the synthesis of acute phase proteins (such as CRP) and promote the production of autoantibodies. On the other hand, it promotes the differentiation of Th2 and Th17 cells while inhibiting the differentiation of Th1 and Treg cells, ultimately leading to an imbalance of Th1/Th2 cells (32, 41). These findings suggest that IL-6 might contribute to CSU pathology, even though the utility of IL-6 as biomarker in CSU is still questionable (42).

### TLR-4 is associated with CSU

Toll-like receptors (TLRs) are a group of pattern recognition receptors that recognise microbe-associated PAMPs and damage or tissue repair hazard-associated sub-DAMPs (43).. TLR-4 was one of the most important TLRs which play a key role in pathogen recognition and activation of various inflammatory signaling pathways (44). TLR-4 signaling also has been suggested to participate in allergic diseases as well as autoimmune responses (45, 46). TLR-4 expression was significantly upregulated in allergic rhinitis patients (47). Lately, although contradictory observations have also been published, TLR-4 polymorphisms have been reported to associate with atopy and asthma (48, 49). Interestingly, a decreased TLR-4 expression on CD14^+^ cells from chronic idiopathic urticaria patients was observed in ex vivo conditions (50). In the present study, for the first time, we identified TLR-4 as a hub gene in CSU. Taken together, these findings indicate that TLR-4 may be involved in the development of CSU. As for the specific mechanisms, clinical sample verification and laboratory data are still needed.

### ICAM-1 might contribute to tissue edema formation in CSU

In CSU, although MCs initiate and remain in early-phase wealing response, cellular adhesion molecules (CAMs) are thought to sustain the late phase (51). CAMs are well documented in the selective recruitment and activation of inflammatory cells, which ultimately lead to tissue damage (52). Among the CAMs, ICAM-1 plays an important role in accelerating tissue oedema formation (53): 1) Overexpression of ICAM-1 in endothelial cells alters cell morphology and increases endothelial cell permeability or vascular leakage (54); 2) Moreover, antibody cross-linking specific for ICAM-1 also increased albumin passage from intact cremaster micro vessels (55). It has been demonstrated that ICAM-1 is the hallmark of allergic inflammation (56). Although it has been reported that ICAM-1 was not always significantly elevated in CU sera. ICAM-1, as a biomarker of endothelial dysfunction, is increased in sera and detected in spontaneous wheals of patients with CIU (35). Moreover, one study, which included a large number of CSU patients, showed a significant increase in soluble ICAM-1 level in sera (57). A recent study also demonstrated that specific knockout of ICAM-1 can inhibits the expression of IgE, ECP, CD62L, and CD11a, thereby reducing the adhesion between vascular endothelial cells in a mouse model of immune contact urticaria (58). In this study, we observed that ICAM-1 was a hub gene in CSU for the first time. Thus, the above previous findings combined with our data, all indicate that ICAM-1 plays an important role in tissue edema formation of CSU, where relevant clinical and laboratory evidence remains to be further explored.

### PTGS-2(COX-2) contributes to non-histamine-mediated symptoms in CSU

In clinic, the effect of using H1 antihistamine alone in the treatment of CSU is not satisfactory (59). A reasonable explanation is that some inflammatory mediators can show similar functions to histamine, such as prostaglandins and coagulans, has been overlooked (60). Arachidonic acid forms prostaglandins and thromboxanes through the actions of the enzymes cyclooxygenases (COXs). Inducible COX-2 is associated with pseudoallergy and other inflammatory reactions (61). Besides, COX-2 is predominantly induced in activated leukocytes and inflammatory sites (62). Meanwhile, Nonsteroidal anti-inflammatory drug (NSAID)-sensitivity is very common in patients with chronic urticaria (63). The physiological and pathological processes of respiratory and skin reactions may involve the increase of cysteinyl leukotrienes (64). COX-2 selective inhibitor is a major alternative treatment to control pain and inflammation in these patients (65). In the present study, we further confirmed PTGS-2 as a hub gene in CSU through PPI network characterization. Thus COX-2 may be an important target for the treatment of CSUs.

### Overview of infiltrating cells in lesional skin of patients with CSU

The study found that CSU lesions contained a higher proportion of DCs, Th2 cells, mast cells, MEP, preadipocytes, and M1 macrophages compared with healthy skin. It was also discovered that increased expression of Th-2 cytokines (IL-4/IL-5) in CSU than in normal healthy controls (66). Mast cells are key effector cells in the pathogenesis of CSU, and when activated, they release inflammatory mediators such as histamine and PGD2, which in turn lead to an increase in vascular permeability, causing flare-ups, itching, angioedema (66). In CSU, IgE-mediated type I hypersensitivity is closely related to mast cell activation (67). A predominance of activated M2 macrophages was also observed in CSU patients with refractory for antihistamines (68). Thus, our experimental data are consistent with previous findings on the overview of immune cell infiltration in the CSU, which in turn demonstrates the accuracy of our analysis.

It’s worth noting that, so far there have been no reports on MEP or preadipocytes in CSU, and these potential connections were observed for the first time in the present study. Thus, the specific mechanism of MEP or preadipocyte involvement in the development of CSU still needs further verification and exploration. Moreover, in this study, we found that hub genes IL-6, TLR-4, ICAM-1, and PTGS-2 were positively correlated with immune and stromal cell infiltration in CSU lesions. These findings indicate that these hub genes also contribute to the infiltration of these immune cells to the lesions of patients with CSU. However, the specific mechanism of these DEGs on the immune invasion of CSU lesions remains to be further studied.

### Limitations of this study

However, this study still has certain limitations. First, the sample size we analyzed is relatively small. Cause the amount of samples uploaded to public databases is currently valid, and we have analyzed the only two databases available until now. Second, all our data comes from publicly available databases. Third, the potential pathway involved in the CSU was not examined and verified. Future studies should be performed to identify the detailed pathway that participated in the progression of CSU. Nevertheless, our results provide a new insight into the pathological mechanism of CSU. These findings do require further substantiation such as by *in vivo* or *in vitro* experiments.

## Conclusions

In summary, in the present study, a total of 4 hub genes (IL-6, TLR-4, ICAM-1, and PTGS-2) have been identified, which has the potential to be used as markers or as therapeutic targets for CSU. TNF signaling pathway, NF-κB signaling pathway, and JAK-STAT signaling pathway are thought to be closely related to the pathological mechanism of CSU. We observed higher proportions of DCs, Th2 cells, mast cells, MEP, preadipocytes, and M1 macrophages in CSU lesions. Finally, the positive correlation of hub genes IL-6, TLR-4, ICAM-1, and PTGS-2 and these immune cells infiltration in CSU lesions were found. Our findings will help to encourage further studies on the biological functions of these hub genes, immune infiltration profiles, as well as the relationship between them in CSU.

## Data availability statement

The datasets presented in this study can be found in online repositories. The names of the repository/repositories and accession number(s) can be found in the article/**Supplementary Material**.

## Author contributions

WS: Software, Visualization, Writing – original draft, Writing – review & editing. YT: Conceptualization, Data curation, Formal Analysis, Writing – review & editing. YW: Data curation, Writing – review & editing. FH: Project administration, Supervision, Writing – review & editing. JJ: Data curation, Project administration, Supervision, Writing – review & editing.

## References

[1] Zuberbier T Abdul Latiff AH Abuzakouk M Aquilina S Asero R Baker D. The international EAACI/GA(2)LEN/EuroGuiDerm/APAAACI guideline for the definition, classification, diagnosis, and management of urticaria. Allergy (2022) 77(3):734–66. doi: 10.1111/all.15090 34536239

[2] Kolkhir P Gimenez-Arnau AM Kulthanan K Peter J Metz M Maurer M. Urticaria. Nat Rev Dis Primers (2022) 8(1):61. doi: 10.1038/s41572-022-00389-z 36109590

[3] Goncalo M Gimenez-Arnau A Al-Ahmad M Ben-Shoshan M Bernstein JA Ensina LF. The global burden of chronic urticaria for the patient and society. Br J Dermatol (2021) 184(2):226–36. doi: 10.1111/bjd.19561 32956489

[4] Kolkhir P Munoz M Asero R Ferrer M Kocatürk E Metz M. Autoimmune chronic spontaneous urticaria. J Allergy Clin Immunol (2022) 149(6):1819–31. doi: 10.1016/j.jaci.2022.04.010 35667749

[5] Maurer M Zuberbier T Metz M. The classification, pathogenesis, diagnostic workup, and management of urticaria: an update. Handb Exp Pharmacol (2022) 268:117–33. doi: 10.1007/164_2021_506 34247278

[6] Zhou B Li J Liu R Zhu L Peng C. The role of crosstalk of immune cells in pathogenesis of chronic spontaneous urticaria. Front Immunol (2022) 13:879754. doi: 10.3389/fimmu.2022.879754 35711438 PMC9193815

[7] Lamot L Vidovic M Perica M Bukovac LT Harjacek M. Microarray and gene expression analysis. Lijec Vjesn (2015) 137(5-6):188–95. 26380479

[8] Desany B Zhang Z. Bioinformatics and cancer target discovery. Drug Discovery Today (2004) 9(18):795–802. doi: 10.1016/S1359-6446(04)03224-6 15364067

[9] Yan Q. Bioinformatics databases and tools in virology research: an overview. In Silico Biol (2008) 8(2):71–85. 18928197

[10] Oertelt S Selmi C Invernizzi P Podda M Gershwin ME. Genes and goals: an approach to microarray analysis in autoimmunity. Autoimmun Rev (2005) 4(7):414–22. doi: 10.1016/j.autrev.2005.05.004 16137606

[11] Patel OP Giorno RC Dibbern DA Andrews KY Durairaj S Dreskin SC. Gene expression profiles in chronic idiopathic (spontaneous) urticaria. Allergy Rhinol (Providence) (2015) 6(2):101–10. doi: 10.2500/ar.2015.6.0124 PMC4541630 26302730

[12] Gimenez-Arnau A Curto-Barredo L Nonell L Puigdecanet E Yelamos J Gimeno R. Transcriptome analysis of severely active chronic spontaneous urticaria shows an overall immunological skin involvement. Allergy (2017) 72(11):1778–90. doi: 10.1111/all.13183 28407332

[13] Metz M Torene R Kaiser S Beste MT Staubach P Bauer A. Omalizumab normalizes the gene expression signature of lesional skin in patients with chronic spontaneous urticaria: A randomized, double-blind, placebo-controlled study. Allergy (2019) 74(1):141–51. doi: 10.1111/all.13547 29974963

[14] Liu R Lv D Cao L Liu Y Wen X Jiang Y. The hub genes and their potential regulatory mechanisms in chronic spontaneous urticaria revealed by integrated transcriptional expression analysis. Exp Dermatol (2023) 32(6):840–51. doi: 10.1111/exd.14785 36856573

[15] Subramanian A Tamayo P Mootha VK Mukherjee K Ebert BL Gillette MA. Gene set enrichment analysis: a knowledge-based approach for interpreting genome-wide expression profiles. Proc Natl Acad Sci U S A (2005) 102(43):15545–50. doi: 10.1073/pnas.0506580102 PMC1239896 16199517

[16] Pathan M Keerthikumar S Ang CS Gangoda L Quek CY Williamson NA. FunRich: An open access standalone functional enrichment and interaction network analysis tool. Proteomics (2015) 15(15):2597–601. doi: 10.1002/pmic.201400515 25921073

[17] Aran D Hu Z Butte AJ. xCell: digitally portraying the tissue cellular heterogeneity landscape. Genome Biol (2017) 18(1):220. doi: 10.1186/s13059-017-1349-1 29141660 PMC5688663

[18] Ferrer M. Immunological events in chronic spontaneous urticaria. Clin Transl Allergy (2015) 5:30. doi: 10.1186/s13601-015-0074-7 26309723 PMC4549074

[19] Church MK Kolkhir P Metz M Maurer M. The role and relevance of mast cells in urticaria. Immunol Rev (2018) 282(1):232–47. doi: 10.1111/imr.12632 29431202

[20] Jang DI Lee AH Shin HY Song HR Park JH Kang TB. The role of tumor necrosis factor alpha (TNF-alpha) in autoimmune disease and current TNF-alpha inhibitors in therapeutics. Int J Mol Sci (2021) 22(5):2719. doi: 10.3390/ijms22052719 33800290 PMC7962638

[21] Doss GP Agoramoorthy G Chakraborty C. TNF/TNFR: drug target for autoimmune diseases and immune-mediated inflammatory diseases. Front Biosci (Landmark Ed) (2014) 19(7):1028–40. doi: 10.2741/4265 24896334

[22] Grzanka R Damasiewicz-Bodzek A Kasperska-Zajac A. Tumor necrosis factor-alpha and Fas/Fas ligand signaling pathways in chronic spontaneous urticaria. Allergy Asthma Clin Immunol (2019) 15:15. doi: 10.1186/s13223-019-0332-7 30911316 PMC6417283

[23] Hermes B Prochazka AK Haas N Jurgovsky K Sticherling M Henz BM. Upregulation of TNF-alpha and IL-3 expression in lesional and uninvolved skin in different types of urticaria. J Allergy Clin Immunol (1999) 103(2 Pt 1):307–14. doi: 10.1016/S0091-6749(99)70506-3 9949323

[24] Sand FL Thomsen SF. TNF-alpha inhibitors for chronic urticaria: experience in 20 patients. J Allergy (Cairo) (2013) 2013:130905. doi: 10.1155/2013/130905 24167521 PMC3791586

[25] Bansal CJ Bansal AS. Stress, pseudoallergens, autoimmunity, infection and inflammation in chronic spontaneous urticaria. Allergy Asthma Clin Immunol (2019) 15:56. doi: 10.1186/s13223-019-0372-z 31528163 PMC6737621

[26] Sur I Ulvmar M Toftgard R. The two-faced NF-kappaB in the skin. Int Rev Immunol (2008) 27(4):205–23. doi: 10.1080/08830180802130319 18574737

[27] Jimi E Fei H Nakatomi C. NF-kappaB signaling regulates physiological and pathological chondrogenesis. Int J Mol Sci (2019) 20(24):6275. doi: 10.3390/ijms20246275 31842396 PMC6941088

[28] Liu T Zhang L Joo D Sun SC. NF-kappaB signaling in inflammation. Signal Transduct Target Ther (2017) 2:17023–. doi: 10.1038/sigtrans.2017.23 PMC5661633 29158945

[29] Luo XY Liu Q Yang H Tan Q Gan LQ Ren FL. OSMR gene effect on the pathogenesis of chronic autoimmune Urticaria via the JAK/STAT3 pathway. Mol Med (2018) 24(1):28. doi: 10.1186/s10020-018-0025-6 30134804 PMC6016876

[30] Feng H Feng J Zhang Z Xu Q Hu M Wu Y. Role of IL-9 and IL-10 in the pathogenesis of chronic spontaneous urticaria through the JAK/STAT signalling pathway. Cell Biochem Funct (2020) 38(4):480–9. doi: 10.1002/cbf.3481 31989663

[31] Hox V O’Connell MP Lyons JJ Sackstein P Dimaggio T Jones N. Diminution of signal transducer and activator of transcription 3 signaling inhibits vascular permeability and anaphylaxis. J Allergy Clin Immunol (2016) 138(1):187–99. doi: 10.1016/j.jaci.2015.11.024 PMC4931983 26948077

[32] Neurath MF Finotto S. IL-6 signaling in autoimmunity, chronic inflammation and inflammation-associated cancer. Cytokine Growth Factor Rev (2011) 22(2):83–9. doi: 10.1016/j.cytogfr.2011.02.003 21377916

[33] Tackey E Lipsky PE Illei GG. Rationale for interleukin-6 blockade in systemic lupus erythematosus. Lupus (2004) 13(5):339–43. doi: 10.1191/0961203304lu1023oa PMC2014821 15230289

[34] Tanaka T Narazaki M Kishimoto T. Therapeutic targeting of the interleukin-6 receptor. Annu Rev Pharmacol Toxicol (2012) 52:199–219. doi: 10.1146/annurev-pharmtox-010611-134715 21910626

[35] Caproni M Volpi W Macchia D Giomi B Manfred M Campi P. Infiltrating cells and related cytokines in lesional skin of patients with chronic idiopathic urticaria and positive autologous serum skin test. Exp Dermatol (2003) 12(5):621–8. doi: 10.1034/j.1600-0625.2003.00010.x 14705803

[36] Metz M Krull C Maurer M. Histamine, TNF, C5a, IL-6, -9, -18, -31, -33, TSLP, neopterin, and VEGF are not elevated in chronic spontaneous urticaria. J Dermatol Sci (2013) 70(3):222–5. doi: 10.1016/j.jdermsci.2013.03.003 23602531

[37] Gora A Przybyl M Swietochowska E Machura E. Assessment of selected interleukins (IL-6, IL-17A, IL-18, IL-23) and chemokines (RANTES, IP-10) in children with acute and chronic urticaria. Ital J Pediatr (2022) 48(1):201. doi: 10.1186/s13052-022-01395-3 36539847 PMC9768875

[38] Grieco T Porzia A Paolino G Chello C Sernicola A Faina V. IFN-gamma/IL-6 and related cytokines in chronic spontaneous urticaria: evaluation of their pathogenetic role and changes during omalizumab therapy. Int J Dermatol (2020) 59(5):590–4. doi: 10.1111/ijd.14812 32048727

[39] de Montjoye L Choteau M Herman A Hendrickx E Chéou P Baeck M. IL-6 and IL-1beta expression is increased in autologous serum skin test of patients with chronic spontaneous urticaria. Allergy (2019) 74(12):2522–4. doi: 10.1111/all.13928 31125442

[40] Kolkhir P Andre F Church MK Maurer M Metz M. Potential blood biomarkers in chronic spontaneous urticaria. Clin Exp Allergy (2017) 47(1):19–36. doi: 10.1111/cea.12870 27926978

[41] Ataie-Kachoie P Pourgholami MH Morris DL. Inhibition of the IL-6 signaling pathway: a strategy to combat chronic inflammatory diseases and cancer. Cytokine Growth Factor Rev (2013) 24(2):163–73. doi: 10.1016/j.cytogfr.2012.09.001 23107589

[42] Asero R Cugno M. Biomarkers of chronic spontaneous urticaria and their clinical implications. Expert Rev Clin Immunol (2021) 17(3):247–54. doi: 10.1080/1744666X.2021.1882304 33496195

[43] Kawai T Akira S. Toll-like receptors and their crosstalk with other innate receptors in infection and immunity. Immunity (2011) 34(5):637–50. doi: 10.1016/j.immuni.2011.05.006 21616434

[44] Lim KH Staudt LM. Toll-like receptor signaling. Cold Spring Harb Perspect Biol (2013) 5(1):a011247. doi: 10.1101/cshperspect.a011247 23284045 PMC3579400

[45] Zhang Y Liu J Wang C Liu J Lu W. Toll-like receptors gene polymorphisms in autoimmune disease. Front Immunol (2021) 12:672346. doi: 10.3389/fimmu.2021.672346 33981318 PMC8107678

[46] Kirtland ME Tsitoura DC Durham SR Shamji MH. Toll-like receptor agonists as adjuvants for allergen immunotherapy. Front Immunol (2020) 11:599083. doi: 10.3389/fimmu.2020.599083 33281825 PMC7688745

[47] Radman M Golshiri A Shamsizadeh A Zainodini N Bagheri V Arababadi MK. Toll-like receptor 4 plays significant roles during allergic rhinitis. Allergol Immunopathol (Madr) (2015) 43(4):416–20. doi: 10.1016/j.aller.2014.04.006 25097025

[48] Shukur W Alyaqubi K Dosh R Al-Ameri A Al-Aubaidy H Al-Maliki R. Association of Toll-like receptors 4 (TLR-4) gene expression and polymorphisms in patients with severe asthma. J Med Life (2021) 14(4):544–8. doi: 10.25122/jml-2021-0173 PMC8485369 34621380

[49] Rylander R Michel O. Organic dust induced inflammation–role of atopy and TLR-4 and CD14 gene polymorphisms. Am J Ind Med (2005) 48(4):302–7. doi: 10.1002/ajim.20205 16142747

[50] Azor MH dos Santos JC Futata EA de Brito CA Maruta CW Rivitti EA. Statin effects on regulatory and proinflammatory factors in chronic idiopathic urticaria. Clin Exp Immunol (2011) 166(2):291–8. doi: 10.1111/j.1365-2249.2011.04473.x PMC3219904 21985375

[51] Caproni M Giomi B Volpi W Melani L Schincaglia E Macchia D. Chronic idiopathic urticaria: infiltrating cells and related cytokines in autologous serum-induced wheals. Clin Immunol (2005) 114(3):284–92. doi: 10.1016/j.clim.2004.10.007 15721839

[52] Quarmby S Kumar P Kumar S. Radiation-induced normal tissue injury: role of adhesion molecules in leukocyte-endothelial cell interactions. Int J Cancer (1999) 82(3):385–95. doi: 10.1002/(SICI)1097-0215(19990730)82:3<385::AID-IJC12>3.0.CO;2-5 10399956

[53] Yanaba K Kaburagi Y Takehara K Steeber DA Tedder TF Sato S. Relative contributions of selectins and intercellular adhesion molecule-1 to tissue injury induced by immune complex deposition. Am J Pathol (2003) 162(5):1463–73. doi: 10.1016/S0002-9440(10)64279-4 PMC1851207 12707029

[54] Lawson C Wolf S. ICAM-1 signaling in endothelial cells. Pharmacol Rep (2009) 61(1):22–32. doi: 10.1016/S1734-1140(09)70004-0 19307690

[55] Sumagin R Kuebel JM Sarelius IH. Leukocyte rolling and adhesion both contribute to regulation of microvascular permeability to albumin via ligation of ICAM-1. Am J Physiol Cell Physiol (2011) 301(4):C804–813. doi: 10.1152/ajpcell.00135.2011 PMC3191564 21653902

[56] Canonica GW Ciprandi G Pesce GP Buscaglia S Paolieri F Bagnasco M. ICAM-1 on epithelial cells in allergic subjects: a hallmark of allergic inflammation. Int Arch Allergy Immunol (1995) 107(1-3):99–102. doi: 10.1159/000236943 7613226

[57] Puxeddu I Panza F Pratesi F Bartaloni D Casigliani Rabl S Rocchi V. CCL5/RANTES, sVCAM-1, and sICAM-1 in chronic spontaneous urticaria. Int Arch Allergy Immunol (2013) 162(4):330–4. doi: 10.1159/000354922 24157824

[58] Lv G Fan J. Silencing ICAM-1 reduces the adhesion of vascular endothelial cells in mice with immunologic contact urticaria. Gene (2020) 760:144965. doi: 10.1016/j.gene.2020.144965 32687948

[59] Zuberbier T Aberer W Asero R Bindslev-Jensen C Brzoza Z Canonica GW. The EAACI/GA(2) LEN/EDF/WAO Guideline for the definition, classification, diagnosis, and management of urticaria: the 2013 revision and update. Allergy (2014) 69(7):868–87. doi: 10.1111/all.12313 24785199

[60] Bristow MR Ginsburg R Kantrowitz NE Baim DS Rosenbaum JT. Coronary spasm associated with urticaria: report of a case mimicking anaphylaxis. Clin Cardiol (1982) 5(3):238–40. doi: 10.1002/clc.4960050307 7083649

[61] Jawabrah Al-Hourani B Sharma SK Suresh M Wuest F. Cyclooxygenase-2 inhibitors: a literature and patent review (2009 - 2010). Expert Opin Ther Pat (2011) 21(9):1339–432. doi: 10.1517/13543776.2011.593510 21714592

[62] Kolaczkowska E Goldys A Kozakiewicz E Lelito M Plytycz B van Rooijen N. Resident peritoneal macrophages and mast cells are important cellular sites of COX-1 and COX-2 activity during acute peritoneal inflammation. Arch Immunol Ther Exp (Warsz) (2009) 57(6):459–66. doi: 10.1007/s00005-009-0053-6 19885646

[63] Stevenson DD. Aspirin and NSAID sensitivity. Immunol Allergy Clin North Am (2004) 24(3):491–505. doi: 10.1016/j.iac.2004.03.001 15242723

[64] Picado P. COX-2 specific inhibitors in NSAID-intolerant patients. Int J Immunopathol Pharmacol (2003) 16(2 Suppl):11–6. 14552699

[65] Vane JR Botting RM. Anti-inflammatory drugs and their mechanism of action. Inflammation Res (1998) 47 Suppl 2:S78–87. doi: 10.1007/s000110050284 9831328

[66] Caproni M Giomi B Melani L Volpi W Antiga E Torchia D. Cellular infiltrate and related cytokines, chemokines, chemokine receptors and adhesion molecules in chronic autoimmune urticaria: comparison between spontaneous and autologous serum skin test induced wheal. Int J Immunopathol Pharmacol (2006) 19(3):507–15. doi: 10.1177/039463200601900306 17026835

[67] Bracken SJ Abraham S MacLeod AS. Autoimmune theories of chronic spontaneous urticaria. Front Immunol (2019) 10:627. doi: 10.3389/fimmu.2019.00627 30984191 PMC6450064

[68] Criado RFJ Criado PR Pagliari C Sotto MN MaChado Filho CD Bianco B. M2 macrophage polarization in chronic spontaneous urticaria refractory to antihistamine treatment. Allergol Int (2021) 70(4):504–6. doi: 10.1016/j.alit.2021.03.005 33994101

[69] Su W Huang B Zhao Y Zhang X Chen L Ji J. (2020). Integrated bioinformatics approach to understand immune-related key genes and pathways in chronic spontaneous urticaria. PREPRINT (Version 1). Available at Research Square: 10.21203/rs.3.rs-137346/v1.

