# Supplementary material for: Key genes and immune infiltration in chronic spontaneous urticaria: a study of bioinformatics and systems biology
Source: Front Immunol. 2023 Nov 15;14:1279139. doi: 10.3389/fimmu.2023.1279139 (PMC10693338; doi:10.3389/fimmu.2023.1279139)
Supplement: Supplementary file 1 [file Table_1.docx]

**Table s1. The top 10 biological processes enriched of DEGs.**

| ID | Term | P-value | Number | Genes |
| --- | --- | --- | --- | --- |
| GO:0006954 | inflammatory response | 4.74E-05 | 9 | IL6, CYBB, PTGS2, FPR1, TLR4, FPR3, FPR2, CCL18, CD14 |
| GO:0050900 | leukocyte migration | 2.32E-04 | 4 | ICAM1, FPR1, FPR3, FPR2 |
| GO:0007155 | cell adhesion | 2.70E-04 | 7 | ICAM1, TNFAIP6, ITGAX, SELL, HAS2, THBS1, CYR61 |
| GO:0043066 | negative regulation of the apoptotic process | 4.90E-04 | 8 | IL6, SOCS3, HCK, DNAJA1, PIM1, GREM1, MYC, CYR61 |
| GO:0045084 | positive regulation of the interleukin-12 biosynthetic process | 8.44E-04 | 3 | IRF1, TLR4 |
| GO:0045087 | innate immune response | 9.73E-04 | 7 | CYBB, S100A8, HCK, S100A9, TLR4, PTX3, CD14 |
| GO:0002430 | complement receptor- mediated signaling pathway | 0.001435 | 3 | FPR1, FPR3, FPR2 |
| GO:0042554 | superoxide anion generation | 0.002173 | 3 | CYBB, NCF2, SOD2 |
| GO:0050729 | positive regulation of inflammatory response | 0.002306 | 4 | S100A8, LDLR, S100A9, CCL18 |
| GO:0030593 | neutrophil chemotaxis | 0.004067 | 4 | S100A8, S100A9, TREM1, CCL18 |
