# Supplementary material for: Key genes and immune infiltration in chronic spontaneous urticaria: a study of bioinformatics and systems biology
Source: Front Immunol. 2023 Nov 15;14:1279139. doi: 10.3389/fimmu.2023.1279139 (PMC10693338; doi:10.3389/fimmu.2023.1279139)
Supplement: Supplementary file 2 [file Table_2.docx]

**Table s2. The KEGG pathway analysis of all the DEGs.**

| ID | Term | P-value | Number | Genes |
| --- | --- | --- | --- | --- |
| hsa04145 | Phagosome | 4.81E-10 | 8 | MRC1, NCF2, TUBA1C, THBS1, CYBB, CD14, TLR4, FCGR2A |
| hsa05144 | Malaria | 4.43E-08 | 5 | IL6, SELE, ICAM1, TLR4, THBS1 |
| hsa04668 | TNF signaling pathway | 7.03E-08 | 6 | PTGS2, IRF1, SOCS3, SELE, IL6, ICAM1 |
| hsa05150 | Staphylococcus aureus infection | 2.05E-07 | 5 | FPR1, FPR2, FPR3, ICAM1, FCGR2A |
| hsa05140 | Leishmaniasis | 3.05E-07 | 5 | CYBB, PTGS2, FCGR2A, TLR4, NCF2 |
| hsa04621 | NOD-like receptor signaling pathway | 9.64E-07 | 6 | IL6, NAMPT, CYBB, IFI16, TLR4, PANX1 |
| hsa05152 | Tuberculosis | 9.95E-07 | 6 | ITGAX, MRC1, FCGR2A, IL6, CD14, TLR4 |
| hsa05167 | Kaposi sarcoma-associated herpesvirus infection | 1.24E-06 | 6 | PTGS2, IL6, ZFP36, ICAM1, HCK, MYC |
| hsa04933 | AGE-RAGE signaling pathway in diabetic complications | 1.26E-06 | 5 | CYBB, SELE, IL6, ICAM1, PIM1 |
| hsa04064 | NF-kappa B signaling pathway | 1.26E-06 | 5 | PTGS2, CD14, CCL13, ICAM1, TLR4 |
| hsa05134 | Legionellosis | 3.76E-06 | 4 | IL6, CD14, HSPA8, TLR4 |
| hsa01100 | Metabolic pathways | 9.02E-06 | 12 | PTGS2, ACER1, MTHFD2, CA2, FAP, UAP1, NAMPT, SRM, PLA2G2A, NNMT, PNP, GFPT2 |
| hsa04630 | Jak-STAT signaling pathway | 1.22E-05 | 5 | IL6, MYC, OSMR, SOCS3, PIM1 |
| hsa05133 | Pertussis | 1.27E-05 | 4 | IL6, CD14, IRF1, TLR4 |
| hsa04657 | IL-17 signaling pathway | 2.72E-05 | 4 | IL6, PTGS2, S100A9, S100A8 |
| hsa00760 | Nicotinate and nicotinamide metabolism | 2.75E-05 | 3 | PNP, NNMT, NAMPT |
| hsa05143 | African trypanosomiasis | 4.93E-05 | 3 | IL6, SELE, ICAM1 |
| hsa04066 | HIF-1 signaling pathway | 4.95E-05 | 4 | CYBB, IL6, TIMP1, TLR4 |
| hsa05166 | Human T-cell leukemia virus 1 infection | 4.97E-05 | 5 | IL6, ETS2, MYC, ZFP36, ICAM1 |
| hsa04380 | Osteoclast differentiation | 9.06E-05 | 4 | TYROBP, NCF2, SOCS3, FCGR2A |
