# Supplementary material for: Key genes and immune infiltration in chronic spontaneous urticaria: a study of bioinformatics and systems biology
Source: Front Immunol. 2023 Nov 15;14:1279139. doi: 10.3389/fimmu.2023.1279139 (PMC10693338; doi:10.3389/fimmu.2023.1279139)
Supplement: Supplementary file 3 [file Table_3.docx]

**Table s3. The pathway analysis of all the DEGs via Reactome database.**

| ID | Term | P-value | Number | Genes |
| --- | --- | --- | --- | --- |
| R-HSA-168256 | Immune System | 1.47E-29 | 39 | PTGS2, MT2A, TIMP1, NCF2, HSPA8, TREM1, PANX1, CD53, MRC1, TYROBP, PTX3, IFI16, OSMR, S100A9, S100A8, ICAM1, MYC, CRISPLD2, FCN1, SELL, PIM1, FPR1, FPR2, IFI30, FCGR2A, CYBB, SERPINB1, HCK, ITGAX, PLA2G2A, IRF1, SOCS3, PNP, TNFAIP6, SLC2A3, IL6, TUBA1C, CD14, TLR4 |
| R-HSA-168249 | Innate Immune System | 1.69E-22 | 26 | NCF2, CD53, HSPA8, TREM1, PANX1, TYROBP, PTX3, IFI16, S100A9, S100A8, CRISPLD2, FCN1, SELL, FPR1, FPR2, FCGR2A, PLA2G2A, SERPINB1, HCK, ITGAX, CYBB, PNP, TNFAIP6, SLC2A3, CD14, TLR4 |
| R-HSA-6798695 | Neutrophil degranulation | 3.55E-20 | 19 | ITGAX, CRISPLD2, FCN1, PTX3, SELL, CD53, TYROBP, HSPA8, FPR2, TNFAIP6, SLC2A3, FCGR2A, CYBB, CD14, S100A9, S100A8, FPR1, SERPINB1, PNP |
| R-HSA-6785807 | Interleukin-4 and Interleukin-13 signaling | 7.65E-13 | 9 | ITGAX, PTGS2, TIMP1, SOCS3, PIM1, HSPA8, IL6, ICAM1, MYC |
| R-HSA-1280215 | Cytokine Signaling in Immune system | 2.79E-11 | 15 | ITGAX, PTGS2, IRF1, MT2A, TIMP1, SOCS3, PIM1, FPR1, HSPA8, IFI30, IL6, OSMR, ICAM1, HCK, MYC |
| R-HSA-449147 | Signaling by Interleukins | 1.31E-09 | 12 | ITGAX, PTGS2, TIMP1, SOCS3, PIM1, FPR1, HSPA8, IL6, OSMR, ICAM1, HCK, MYC |
| R-HSA-196854 | Metabolism of vitamins and cofactors | 2.21E-09 | 8 | PTGS2, MTHFD2, VNN2, SLC2A3, NAMPT, LDLR, NNMT, SLC5A6 |
| R-HSA-8953897 | Cellular responses to external stimuli | 2.55E-09 | 11 | NCF2, DNAJA1, MT2A, HSPH1, MT1M, TUBA1C, HSPA8, CYBB, IL6, ETS2, MT1A |
| R-HSA-196849 | Metabolism of water-soluble vitamins and cofactors | 3.10E-09 | 7 | PTGS2, MTHFD2, VNN2, SLC2A3, NAMPT, NNMT, SLC5A6 |
| R-HSA-1280218 | Adaptive Immune System | 1.04E-08 | 12 | SELL, NCF2, TLR4, SOCS3, MRC1, TYROBP, IFI30, CYBB, TUBA1C, CD14, TREM1, ICAM1 |
| R-HSA-6783783 | Interleukin-10 signaling | 3.65E-08 | 5 | IL6, PTGS2, TIMP1, ICAM1, FPR1 |
| R-HSA-5686938 | Regulation of TLR by endogenous ligand | 7.69E-08 | 4 | CD14, S100A9, S100A8, TLR4 |
| R-HSA-5668599 | RHO GTPases Activate NADPH Oxidases | 1.77E-07 | 4 | CYBB, NCF2, S100A9, S100A8 |
| R-HSA-444473 | Formyl peptide receptors bind formyl peptides and many other ligands | 6.23E-07 | 3 | FPR1, FPR2, FPR3 |
| R-HSA-1430728 | Metabolism | 7.35E-07 | 16 | PTGS2, ACER1, MTHFD2, NNMT, VNN2, CA2, SLC2A3, NAMPT, SRM, PLA2G2A, LDLR, ALOX5AP, SLC5A6, PNP, HAS2, CH25H |
| R-HSA-877300 | Interferon gamma signaling | 7.67E-07 | 5 | IRF1, SOCS3, MT2A, IFI30, ICAM1 |
| R-HSA-2262752 | Cellular responses to stress | 9.95E-07 | 8 | NCF2, DNAJA1, HSPH1, TUBA1C, HSPA8, CYBB, IL6, ETS2 |
| R-HSA-1236975 | Antigen processing-Cross presentation | 1.15E-06 | 5 | CYBB, NCF2, CD14, TLR4, MRC1 |
| R-HSA-109582 | Hemostasis | 1.71E-06 | 9 | ITGAX, SELL, IRF1, TIMP1, TUBA1C, SELE, THBS1, TREM1, SERPINE2 |
| R-HSA-5661231 | Metallothioneins bind metals | 9.06E-05 | 3 | MT1M, MT2A, MT1A |
